# Changes in traditional ecological knowledge of forage plants in immigrant villages of Ningxia, China

**DOI:** 10.1186/s13002-019-0333-0

**Published:** 2019-12-16

**Authors:** Ying Ma, Binsheng Luo, Qiang Zhu, Dongxing Ma, Qi Wen, Jinchao Feng, Dayuan Xue

**Affiliations:** 1 College of Life and Environmental Sciences, Minzu University of China, Beijing, 100081 People’s Republic of China; 2 Ningxia Forestry Research Institute, Yinchuan, 750021 Ningxia People’s Republic of China; 3 College of Mathematics and Information Science, North Minzu University, Yinchuan, 750021 Ningxia People’s Republic of China; 4 College of Resources and Environment, Ningxia University, Yinchuan, 750021 Ningxia People’s Republic of China

**Keywords:** Ecological immigrant, Forage plant, Traditional knowledge, Hongsibu District, Ningxia

## Abstract

**Background:**

Ecological migration serves as an important measure for poverty eradication as well as for the protection, inheritance, and utilization of traditional ecological knowledge. This study investigated and cataloged the traditional forage plant resources and recorded the associated traditional knowledge of immigrant villages in Hongsibu District of Ningxia, China. The diversity of traditional forage plant resources and the changes in associated traditional ecological knowledge were compared among ecological immigrant villages from different emigration areas, with a hope of providing a reference for forage development, the conservation of wild forage plant resources, and the development of regional animal husbandry.

**Methods:**

From March 2018 to May 2019, a field investigation was conducted in six villages in Ningxia. Through the snowball technique, a total of 315 immigrants were interviewed using various methods, including semistructured interviews and key person interviews, which included opportunities for free listing. The changes in the utilization of traditional forage plants were compared between the ecological migrants and the original inhabitants, and the causes underlying the changes were analyzed. In addition, the major forage plant species in the research area were investigated and evaluated.

**Results:**

(1) The six investigated villages reported 224 traditional forage plant species that belong to 42 families and 150 genera. Compared with their original living areas, the number of traditional forage plant species used in the immigrant villages decreased with the increase in the relocation distance. (2) The utilization of traditional forage plants varied among the immigrants who moved to Hongsibu District from forest areas, loess hilly areas, and semiarid desertified areas. The smaller the difference was in ecological environment between the immigration and emigration areas, the more the traditional forage plant knowledge had been retained. (3) The diversity and associated knowledge of traditional forage plants retained by ecological migrants are closely correlated to gender, age, education level, and occupation.

**Conclusion:**

This study revealed that the diversity of traditional forage plants and associated knowledge retained after migration vary among ecological immigrants from different areas; generally, the immigrants that relocated from a closer place retained more ecological knowledge. In the immigrant villages with significantly different natural resources and a long distance from the migrants’ original locations, the diversity of traditional forage plants decreased, and the traditional knowledge about forage plants showed signs of being forgotten and abandoned by the younger generation. Therefore, measures are urgently needed to document and protect the forage plant resources and preserve the traditional knowledge of ecological immigrants.

## Background

Based on the characteristics of local plant resources, local farmers and herdsmen inherit traditional knowledge of forage plants [1] and have a deep understanding of and practical experience with local forage resources [2]. This plays an important role in maintaining the positive development of agricultural and animal husbandry socioecological systems [3]. Local farmers’ and herdsmen’ traditional knowledge of forage plants, gained from local livelihoods and cultures that rely on natural herbage [4], plays an important role in regional biodiversity protection [5]. Farmers and herdsmen in areas with a long history of rearing livestock have a rich knowledge of forage plants, which has been recorded in the countries such as Morocco, West Africa, Brazil, Ethiopia, Pakistan, and Austria [2, 4, 6–10] to provide techniques and strategies for utilization, classification management, and sustainable development of forage plant resources. As a contracting party of the Convention on Biological Diversity, China has made active efforts to protect and record traditional knowledge associated with biodiversity and biological resources over the past two decades [11, 12]. Traditional knowledge for sustainable use of forage plants is indispensable and invaluable. For example, under China’s policy of returning farmland to forests and grassland, farmers and herdsmen of Dulong (Drung or T’rung, an ethnic group in China) in the area of Yunnan Province, where available grassland resources are very limited, successfully maintained a sustainable agricultural system for livestock, forage plants, and crops by using their knowledge of native forage plants [7]. Animal husbandry is an ecological adaptation of humans living in grasslands. The herdsmen in Inner Mongolia of China have accumulated relevant knowledge regarding palatability and seasonal periodicity of forage plants, as well as livestock fatness status, which provides a good reference for the modernization of animal husbandry, such as intensive animal husbandry and the development of family farms [13].

Ecological migration is a phenomenon of population migration due to the interaction of ecological environment and other factor s[14–16]. In China, due to the needs of ecological civilization construction [17] and the aim of eliminating poverty [18], populations formerly living in those areas for designed nature preservation, or with severely damaged ecological environments, or ecologically fragile, or not suitable for human habitation, are requested to emigrate to other places for settlement. This is called ecological migration [14–18]. Ecological migration plays an important role in effectively alleviating environmental degradation [19], improving the living standards of immigrants, and developing local economy [20]. As an important measure of protecting biodiversity and reducing poverty [21], ecological migration results in a win-win situation to a certain extent. Ecological migration is of great value for preserving traditional cultures and knowledge related to biodiversity [22–25]. However, the separation of immigrants from their original natural resources and cultural atmosphere brings serious challenges regarding the preservation of traditional knowledge accumulated over generations [25], especially the inheritance of farmers and herdsmen’s traditional knowledge of forage plants.

Ningxia is representative of an agro-pastoral transition zone [26] in China with massive ecological migration [27], where a thorough understanding regarding the connection and change between migrants and forage plant knowledge is particularly important. The forage plants in different ecological regions such as loess hilly regions, forest regions, and semiarid desertified regions in Ningxia have different characteristics. Therefore, traditional forage plant knowledge accumulated by farmers and herdsmen is regional, dependent, and adaptable. However, little attention has been paid to the impact of ecological migration on forage plant knowledge, and related research findings are scarce. Hence, further research on this topic is urgently needed. Through investigating the retention of traditional knowledge regarding forage plants and related livestock feeding experiences among ecological immigrants from different emigration areas in the Hongsibu District of Ningxia, this study aims to quantitatively analyze the changes in forage plant resources and the underlying influencing factors. Our findings may provide reference for the protection of forage plant resources and associated traditional knowledge during the implementation of ecological migration policy.

## Method

### Research area

**Fig. 1:**
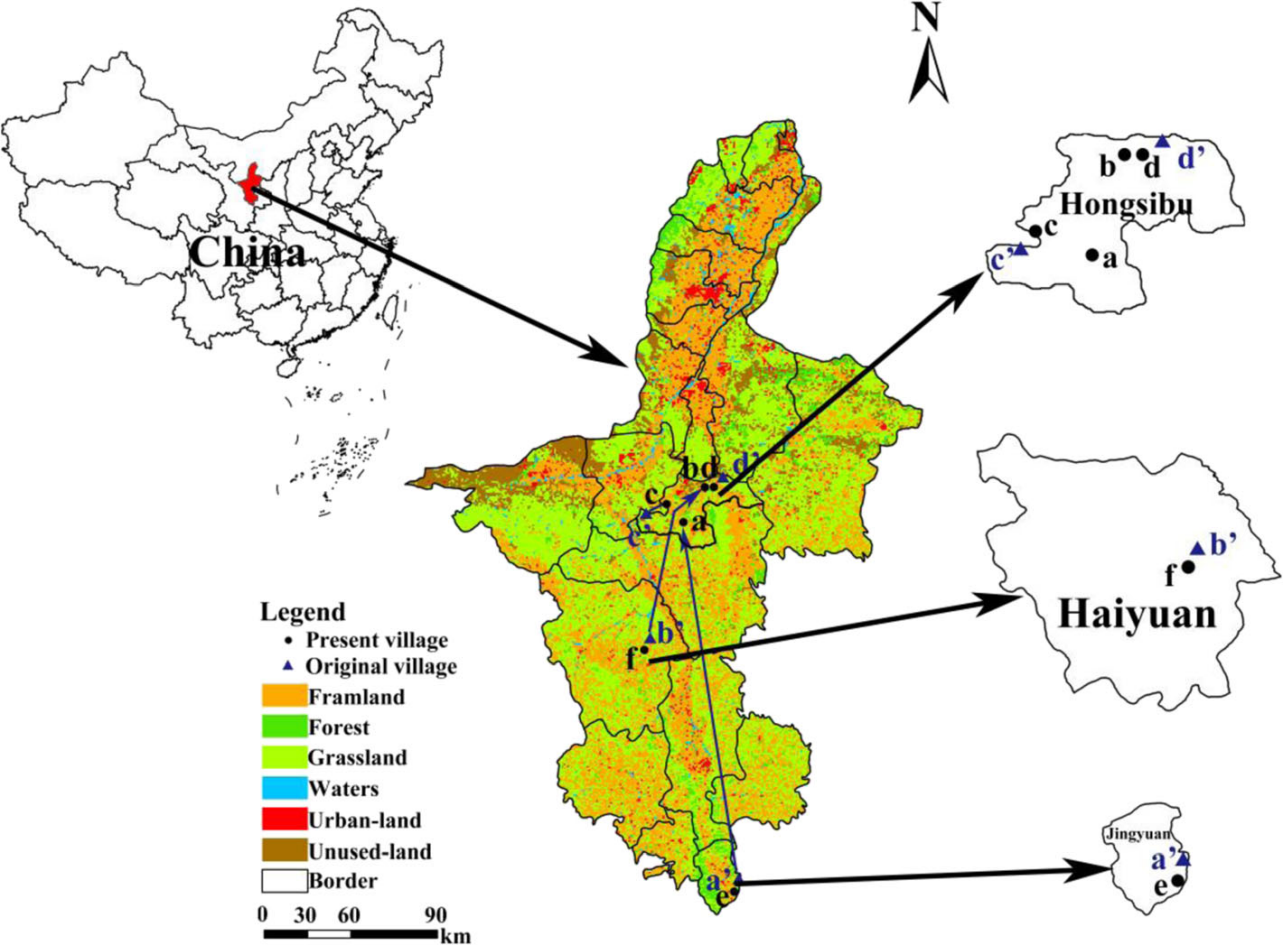
Distribution map of the surveyed villages

The research was conducted in six villages in the Ningxia Hui Autonomous Region: four immigration villages in Hongsibu District, one emigration village in Jingyuan County, Guyuan Prefecture, and one emigration village in Haiyuan County, Zhongwei Prefecture (Fig. 1, Tables 1 and 2).

**Table 1:** Geo-relations of six villages in the research area

Ecological immigrant type	Ecological immigration location	Migration distance	Migration date	Neighboring village of the emigration area
Immigrants from the Liupan Mountain water conservation area	Village a, Liushutai Village Group 2 in Xinzhuang, inhabited by people who moved from Shangwan Village in Xinmin, Jingyuan County, Guyuan	Approximately 300 km	2006	Village e: Zhangtai Village Team 3 in Xinmin, Jingyuan County, Guyuan, which is an unmoved neighboring village of the original Shangwan Village in Xinmin, Jingyuan County, Guyuan
Immigrants from loess hilly areas	Village b: Yongxin Village Xinquan Group in Liuquan, inhabited by people who moved from Talachuan in Hechuan Village, Jiatang, Haiyuan County, Zhongwei	Approximately 200 km	1999	Village f: Chenwan Team of Baotai Village in Jiatang, Haiyuan County, Zhongwei, which is an unmoved neighboring village of the original Talachuan natural village of Hechuan Village in Jiatang, Haiyuan County, Zhongwei
Immigrants from key arid and sandstorm control areas under dryland–to–nearby wetland migration projects	Village c: Xiangyuan Village Xiangyuan Group in Dahe, inhabited by people who moved from Shuayishan Village in Shitangou Town, Tongxin County, Wuzhong	Approximately 20 km	2000	No residents near the emigration area
Immigrants from key arid and sandstorm control areas under dryland–to–nearby wetland migration projects	Village d: Shuitao Village Group 1 in Liuquan, inhabited by people who moved from Shuitao Village - Wanghutai Village in Weizhou, Tongxin County, Wuzhong	Approximately 2 km	2000	No residents near the emigration area

The dryland-to-nearby wetland migration projects resettle immigrants from deep mountain areas lacking water sources and transportation to nearby flat areas with water sources, with the migration distance less than 20 km

Neighboring village of the emigration area refers to the village that remains in the vicinity of the immigrants’ original residence

**Table 2:** Basic information of the six villages in the research area

The village	Number of households	Population	Number of households interviewed	Interview proportion	Altitude (m)	Geographic location
Village a	118	560	42	36%	1475	106° 10′ E, 37° 27′ N
Village b	101	370	39	39%	1300	106° 24′ E, 37° 46′ N
Village c	240	820	73	30%	1300	105° 99′ E, 37° 37′ N
Village d	180	730	68	38%	1263	106° 29′ E, 37° 46′ N
Village e	128	538	48	38%	1821	106° 47′ E, 35° 36′ N
Village f	120	397	45	38%	1593	105° 86′ E, 36° 60′ N

Hongsibu District of Wuzhong, Ningxia, is located in the hinterland of the arid zone in central Ningxia (between 105° 43′ and 106° 42′ E, 37° 28′ and 37° 37′ N). The region is predominantly sandlands and grasslands in the grassland desertification control area in central Ningxia (Fig. 1). It has a dry climate with annual precipitation of 200–400 mm. It is mainly covered by psammophytic steppe and desert steppe. In 1998, supported by the national Seven-Year Priority Poverty Reduction Program, the Yellow River Irrigation Project for Poverty Reduction in Ningxia (“1236” Project) was launched. As a result, Hongsibu District was formed by pumping water from the Yellow River to irrigate the barren land. Within 20 years, more than 200,000 people in central and southern Ningxia moved into Hongsibu District, an ecological migration representative area of poverty-alleviation actions. The four villages in the Hongsibu District research area were Liushutai Village Group 2 in Xinzhuang, inhabited by people who moved from Shangwan Village in Xinmin, Jingyuan County, Guyuan, in 2006 (Village a); Yongxin Village Xinquan Group in Liuquan, inhabited by people who moved from Talachuan in Hechuan Village, Jiatang, Haiyuan County, Zhongwei, in 1999 (Village b); Xiangyuan Village Xiangyuan Group in Dahe, inhabited by people who moved from Shuayishan Village in Shitangou Town, Tongxin County, Wuzhong, in 2000 (Village c); and Shuitao Village Group 1 in Liuquan, inhabited by people who moved from Shuitao Village–Wanghutai Village in Weizhou, Tongxin County, Wuzhong, in 2000 (Village d). Among them, Villages c and d are semiarid desertified areas, and their immigrants migrated from areas within 5 km.

Jingyuan County of Guyuan (106° 12′–106° 29′ E, 35° 15′–35° 37′ N), located in the Liupan Mountain water conservation area in southwestern Ningxia, is the origin of the Jing River, Qingshui River, and Hulu River. It has a relatively humid climate with an annual precipitation of 300–600 mm. This area is covered with diverse types of vegetation, including temperate deciduous broad-leaved forests, coniferous and broad-leaved mixed forests, mountain shrub steppe, and alpine and subalpine meadows. Animal and plant species in the area are abundant, offering an important gene pool of animal and plant resources in Ningxia. In this research area was Village e: Zhangtai Village Team 3 in Xinmin, Jingyuan County, Guyuan, which is within a forest area. This village neighbors the location from where the residents of Village a emigrated. Haiyuan County of Zhongwei (105° 09′–106° 10′ E, 36° 06′–37° 04′ N) is located in the loess hilly area of Ningxia, which mainly includes loess hilly-gully and soil-rock mixed mountainous areas. It has an annual precipitation of 300–500 mm and is mainly covered by artificial deciduous broad-leaved forests, forest grasslands, and steppe. In this research area was Village f: Chenwan Team of Baotai Village, Jiatang of Haiyuan County, Zhongwei. It is a loess hilly area that neighbors the location from where the residents of Village b emigrated.

The dominant contributor to the livelihood of the six villages included in this study is animal husbandry, mainly cattle, and sheep farming. Other contributors include crop and forage production, forestry management, and related sectors. The ecological environment of these villages in Ningxia can be divided into three types: semiarid desertified for Villages a, b, c, and d; forest for Village e; and loess hilly for Village f. Correspondingly, the types of forage plant resources vary among the three ecological environment types. Further, considering the migration status, the six villages selected are ideal for assessing changes in traditional forage plant knowledge of ecological migrants. In this study, the impact of ecological migration on the retention of traditional forage plant knowledge was analyzed through evaluating the similarities and differences between migrants and residents in the unmoved village neighboring the original emigration location (Tables 1 and 2).

### Data collection

Data were collected from March 2018 to May 2019. During the initial stage from March to May 2018, a preliminary investigation of ecological migration projects and forage plant resources in Ningxia was conducted, from which six representative villages were selected for data collection. Subsequently, field surveys were conducted in the six villages from June 2018 to May 2019. With the help of general secretaries of local village committees, heads of forest and grassland stations, and local livestock farming masters (“Yang ba shi”), 315 participants (165 males and 150 females) were sampled through a snowballing technique and given semistructured interviews. The interviewed households accounted for approximately 30% of total households in each village [7, 9, 28]. Prior to the study, informed consent was obtained from all participants regarding the interview procedures. The demographic characteristics, including gender, age, education level, and occupation, are presented in Table 3 [4, 8].

**Table 3:** Sociodemographic characteristics of participants

Category	Gender		Age				Education Level				Occupation						
	Male	Female	≤ 19	20–39	40–59	≥ 60	I	P	M	H	V	F	B	W	D	S	O
Village a	21	21	4	10	18	10	13	22	4	3	3	32	0	1	1	4	1
Village b	21	18	4	13	17	5	16	16	4	3	1	27	1	6	0	3	1
Village c	42	31	5	13	38	17	27	32	11	3	3	61	0	2	1	5	1
Village d	27	41	6	24	21	17	31	19	16	2	2	57	2	3	0	3	1
Village e	26	22	6	11	19	12	18	16	9	5	1	31	2	4	3	5	2
Village f	28	17	3	14	14	14	23	14	5	3	2	36	0	3	0	3	1
Quantity	165	150	28	85	127	75	128	119	49	19	12	244	5	19	5	23	7
Proportion	52%	48%	9%	27%	40%	24%	40%	38%	16%	6%	4%	77%	2%	6%	2%	7%	2%

Education level: I = Illiteracy; P = primary school; M = middle high school; H = High school. Occupation: V = village heads; F = farmers; B = businessmen; W = migrant workers; D = village doctors; S = students; O = others

In the field surveys, respondents were invited to freely list the forage plants they used [8, 29]. The interview included the following questions: (1) Do you know which plants cattle and sheep like to eat? (2) What plants do you feed your cattle and sheep? (3) What parts of these plants do cattle and sheep eat? (4) Are these plants fed to cattle and sheep as tender grass or stored green hay? (5) What types of plants are used to feed livestock in each season? (6) Is there any difference in the plants fed to cattle and sheep before and after migration?

In the second stage of the field study, accompanied by plant experts from the local forest and grassland station and by livestock farming masters, the investigators identified and collected forage plant samples and documented the detailed information of each sample. Voucher specimens and physical samples of forage plants were collected according to various identification documents, including *Flora of China*, *Flora of Ningxia*, *Flora of Liupan Mountain*, *Atlas of Animals and Plants of Nanhua Mountain in Ningxia*, and *Vascular Plants in Luoshan Mountain of Ningxia*. All voucher specimens were stored in the College of Life and Environmental Sciences, Minzu University of China. After collection and preliminary identification, the plant samples were identified and verified by plant taxonomic experts, including Qiang Zhu from the Ningxia Forestry Research Institute, Bo Liu from Minzu University of China, Sheng Qiang from Nanjing Agricultural University, and Kechang Li, a former head of a forest and grassland station in Ningxia.

### Data analysis

Traditional forage plant information collected from 315 respondents was analyzed using four data analysis methods.

1. Relative frequency of citation (RFC): $RFC=\frac{FCs}{N}$

This parameter refers to the ratio of the number of respondents who mention a particular forage plant (i.e., frequency of citation, FC) to the number of all respondents participating in the survey (*N*). The larger the RFC, the more important and valuable the forage plant is in the area. The importance of each forage plant was indicated by its FC value, which allowed all forage plants mentioned in the survey to be listed in order of importance [30, 31].

2. Jaccard $JI=\frac{C}{A+b-C}\times 100$ index (JI):

where *A* represents the number of forage plant species used by villagers in Village a, *B* represents the number of forage plant species used by villagers in Village b, and *C* represents the number of forage plant species used by both Village a and Village b. The JI can be used to compare and evaluate the similarity in the use of forage plant species between two villages [32]. The JI value is between 0 and 100. The greater the value is, the more similar two villages are in using forage plants; low values indicate that there is a great difference in the understanding and utilization of plants between the two villages [33, 34].

3. Cluster analysis

The types of forage plants used by the villagers were recorded and counted for analysis with each village as a unit. Using the chi-square value of the number of forage plant types used in each village as a distance function, systematic cluster analysis was performed to evaluate the similarity in the use of forage plants between villages by using an intergroup classification method. The greater the similarity is, the smaller the distance function. Subsequently, a classification was conducted based on distance function. Cluster analysis was used as a supplement to the JI-based assessment to verify the similarities and differences in the diversity of traditional forage plants used by villagers from different villages.

4. Multivariate analysis of variance (MANOVA)

Four factors (gender, age, education level, and identity/occupation of the respondents) were used as reference variables. MANOVA was performed to evaluate whether the four reference variables had a significant impact on the number of forage plants mentioned by the respondents.

## Results

### Diversity and distribution of traditional forage plants

**Fig. 2:**
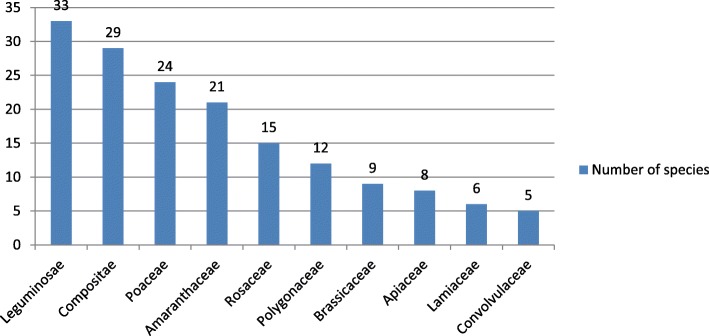
Plant families that include at least five forage plant species in the research area

A total of 224 forage plant species belonging to 150 genera in 42 families were enumerated by respondents during the survey (Table 4). The following are the plant families that included more than ten traditional forage plant species: Leguminosae (33 species, 14.10%), Compositae (29 species, 12.39%), Gramineae (24 species, 10.26%), Amaranthaceae (21 species, 8.97%), Rosaceae (15 species, 6.41%), and Polygonaceae (12 species, 5.13%) (Fig. 2). Among 224 forage plant species, 181 (81%) are herbaceous and lianoid plants, and 43 (19%) are trees and shrubs, suggesting that the local farmers and herdsmen mainly depend on herbaceous plants as livestock feed. For most traditional forage plants (213 species, 95%), stems and leaves are the most common parts used for livestock feed; among them, for 39 species (17.4%), flowers and fruit were also used for livestock feed. This indicates a high diversity of local forage plant resources. For 11 species (0.05%), only roots, flowers, and seeds can be used for livestock feed. Although these forage plants only account for a small proportion, each has its own unique characteristics and should not be ignored. For example, in two plant species (0.008%), *Potentilla anserina* and *Daucus Carota*, roots are the only part used for livestock feed, but they are highly favored by cattle and sheep due to a rich starch content and juicy taste. In three species (0.013%), *Schisandra chinensis*, *Sophora alopecuroides*, and *Eruca vesicaria*, only the fruit is used for livestock feed. The fruit of *S*. *chinensis*, which has a sour, “numbing,” and spicy taste, can be used as medicine. For *S*. *alopecuroides*, only the seeds can be fed to sheep and cattle, but they are seasonally toxic, thus requiring more caution when used as feed. *E. vesicaria* is a local oil crop, and its oil cake and oil residue are nutritious livestock feed. For two plant species (0.008%), *Artemisia argyi* and *Ammopiptanthus mongolicus*, only the flowers can be used for livestock feed. The former is mainly used for medicinal moxibustion in local areas, but its inflorescence is occasionally used to feed cattle and sheep. For *A. mongolicus*, only sheep eat some flowers.

**Table 4:** Traditional forage plants and their relative frequency of citation (RFC)

Family name	Scientific name	Chinese name	Local name	Life form	Part used	Frequency of citations in the surveyed villages						Frequency of citation (FC)	Relative frequency of citation (RFC)
						a	b	c	d	e	f		
Schisandraceae	*Schisandra chinensis* (Turcz.) Baill.	Wu Wei Zi	Chan tiao wan, Ye pu tao wan	Liana	Fruit	0	0	0	0	2	0	2	0.63
Xanthorrhoeaceae	*Hemerocallis citrina* Baroni	Huang Hua Cai	Huang hua cai, Jin zhen hua	Herb	Stem, leaf	0	0	32	0	0	0	32	10.16
Amaryllidaceae	*Allium mongolicum* Regel	Meng Gu Jiu	Sha cong	Herb	Stem, leaf	0	0	53	15	0	1	69	21.9
Amaryllidaceae	*Allium polyrhizum* Turcz. ex Regel	Jian Jiu	Shi cong	Herb	Stem, leaf	0	0	25	15	0	0	40	12.7
Amaryllidaceae	*Allium tenuissimum* L.	Xi Ye Jiu	Yang hu zi	Herb	Stem, leaf	0	0	0	0	0	28	28	8.89
Typhaceae	*Typha domingensis* Pers.	Chang Bao Xiang Pu	Mao la	Herb	Stem, leaf	0	0	0	0	0	6	6	1.9
Poaceae	*Agropyron cristatum*	Bing Cao	Bing cao, Bi dang zi, Huang shu yi ba	Herb	Stem, leaf	35	23	57	54	36	38	243	77.14
Poaceae	*Avena sativa* L.	Yan Mai	Da yan mai, Da yan, Huo yan mai	Herb	Stem, leaf	2	0	0	0	33	26	61	19.37
Poaceae	*Calamagrostis epigejos* (L.) Roth.	Fu Zi Mao	Lu cao	Herb	Stem, leaf	0	0	3	39	0	30	72	22.86
Poaceae	*Chloris virgata* Sw.	Hu Wei Cao	Gou wei cao, Ba ban cao	Herb	Stem, leaf	3	4	20	1	0	0	28	8.89
Poaceae	*Cleistogenes squarrosa* (Trin.) Keng	Zao Yin Zi Cao	Xuan feng cao	Herb	Stem, leaf	0	7	45	34	0	23	109	34.6
Poaceae	*Echinochloa crus-galli* (L.) P.Beauv.	Bai	Bai zi cao, Bing cao	Herb	Stem, leaf, seed	35	12	3	49	36	38	173	54.92
Poaceae	*Eragrostis ferruginea* (Thunb.) P.Beauv.	Xiao Hua Mei Cao	Xiang mao zi, Xiang mao	Herb	Stem, leaf	0	4	59	52	0	30	145	46.03
Poaceae	*Koeleria pyramidata* (Lam.) P.Beauv.	Da Cao	Bing cao, Liu yue he	Herb	Stem, leaf	0	0	0	0	36	0	36	11.43
Poaceae	*Leymus secalinus* (Georgi) Tzvelev	Lai Cao	Bing cao	Herb	Stem, leaf	0	0	0	0	0	38	38	12.06
Poaceae	*Panicum miliaceum* L.	Mi Zi	Mi zi cao, Hong mi zi, Huang mi zi	Herb	Stem, leaf	0	0	0	16	0	41	57	18.1
Poaceae	*Pennisetum centrasiaticum* Tzvelev	Zhong Ya Bai Cao	bia cao, Lang wei cao, Dao sheng zi	Herb	Stem, leaf	0	9	3	18	0	30	60	19.05
Poaceae	*Phragmites australis* (Cav.) Trin. ex Steud.	Lu Wei	Lu nia, Lu cao	Herb	Stem, leaf	0	0	0	0	19	0	19	6.03
Poaceae	*Secale cereale* L.	Hei Mai	Ye yang mai	Herb	Stem, leaf	0	0	0	0	31	0	31	9.84
Poaceae	*Setaria italica* (L.) P.Beauv.	Gu Zi	He cao, Gu cao	Herb	Stem, leaf	0	0	0	0	0	35	35	11.11
Poaceae	*Setaria viridis* (L.) P.Beauv.	Gou Wei Cao	Gu you zi, Mao er gu zhu	Herb	Stem, leaf	5	15	63	59	35	35	212	67.3
Poaceae	*Sorghum bicolor* (L.) Moench	Gao Liang	Gao liang, Chu chu	Herb	Stem, leaf	0	1	0	0	27	17	45	14.29
Poaceae	*Stipa tianschanica* Roshev.	Ge Bi Zhen Mao	Suo cao	Herb	Stem, leaf	0	0	53	58	0	36	147	46.67
Poaceae	*Stipa breviflora* Griseb.	Duan Hua Zhen Mao	Suo cao	Herb	Stem, leaf	0	0	53	58	0	36	147	46.67
Poaceae	*Stipa bungeana* Trin.	Chang Mang Cao	Suo cao	Herb	Stem, leaf	0	0	53	58	0	36	147	46.67
Poaceae	*Stipa caucasica* Schmalh.	Sha Sheng Zhen Mao	Suo cao	Herb	Stem, leaf	0	0	53	58	0	36	147	46.67
Poaceae	*Stipa grandis* P.A.Smirn.	Da Zhen Mao	Suo cao	Herb	Stem, leaf	0	0	53	58	0	36	147	46.67
Poaceae	*Stipa splendens* Trin.	Ji Ji Cao	Ji ji, Xi ji hu zi, Suan cao	Herb	Stem, leaf	2	6	4	6	20	39	77	24.44
Poaceae	*Triticum aestivum* L.	Xiao Mai	Hong mang dong mai, Mia cao, Hong mang chun mai	Herb	Stem, leaf	0	0	33	0	0	35	68	21.59
Poaceae	*Zea mays* L.	Yu Mi	Yu mi	Herb	Stem, leaf	12	18	47	45	48	9	179	56.83
Berberidaceae	*Berberis brachypoda* Maxim.	Duan Bing Xiao Bo	Suan bu liu shu	Shrub	Leaf	0	0	0	0	8	0	8	2.54
Ranunculaceae	*Actaea asiatica* H.Hara	Lei Ye Sheng Ma	Mi la de gan	Herb	Stem, leaf	0	0	0	0	10	0	10	3.17
Ranunculaceae	*Actaea cimicifuga* L.	Sheng Ma	Sheng ma	Herb	Stem, leaf	0	0	0	0	5	0	5	1.59
Ranunculaceae	*Thalictrum petaloideum* L.	Ban Rui Tang Song Cao	Nai de cao, Yang nai cao, Yang nai de hua	Herb	Leaf	0	0	0	0	22	0	22	6.98
Grossulariaceae	*Ribes maximowiczianum* Komarov	Jian Ye Cha Biao Zi	Cha ye mu	Shrub	Leaf	0	0	0	0	3	0	3	0.95
Zygophyllaceae	*Tribulus terrestris* L.	Ji Li	Ba jue zi	Herb	Stem, leaf	5	3	63	57	0	30	158	50.16
Leguminosae	*Ammopiptanthus mongolicus* (Kom.) S.H.Cheng	Sha Dong Qing	Dong qing	Shrub	Flower	0	0	8	0	0	0	8	2.54
Leguminosae	*Astragalus efoliolatus* Hand.-Mazz.	Dan Ye Huang Qi	Mao ti ti hua	Herb	Stem, leaf	0	0	4	8	0	3	15	4.76
Leguminosae	*Astragalus scaberrimus* Bunge	Cao Ye Huang Qi	Mao ti ti hua	Herb	Stem, leaf	0	0	0	0	0	3	3	0.95
Leguminosae	*Astragalus propinquus* Schischkin	Huang Qi	Huang qi	Herb	Stem, leaf	0	0	0	0	3	2	5	1.59
Leguminosae	*Caragana korshinskii* Kom.	Ning Tiao Jin Ji Er	Niu ban jing cao	Shrub	Stem, leaf	11	2	11	37	0	28	89	28.25
Leguminosae	*Caragana stenophylla* Pojark.	Xia Ye Jin Ji Er	Niu ban jing ci	Shrub	Stem, flower, fruit	0	0	3	2	0	28	33	10.48
Leguminosae	*Caragana tibetica* Kom.	Mao Ci Jin Ji Er	Tie mao tou, Hei mao tou, Da mao tou	Shrub	Stem, leaf, flower	0	0	60	39	0	27	126	40
Leguminosae	*Glycyrrhiza uralensis* Fisch.	Gan Cao	Gan cao	Herb	Stem, leaf, fruit	26	6	2	8	0	37	79	25.08
Leguminosae	*Gueldenstaedtia stenophylla* Bunge	Xia Ye Mi Kou Dai	Mi gu zhuang zhuang, Liang shi zhuang zhuang	Herb	Leaf, flower, fruit	0	0	43	19	1	0	63	20
Leguminosae	*Gueldenstaedtia verna* (Georgi) Boriss.	Shao Hua Mi Kou Dai	Mi gu zhuang zhuang	Herb	Stem, leaf	0	0	0	0	2	0	2	0.63
Leguminosae	*Lathyrus davidii* Hance	Da Shan Li Dou	Jing er wan	Herb	Stem, leaf	0	0	0	0	21	0	21	6.67
Leguminosae	*Lathyrus quinquenervius* (Miq.) Litv.	Shan Li Dou	Jing er wan	Herb	Stem, leaf	0	0	0	0	20	0	20	6.35
Leguminosae	*Lens culinaris* Medik.	Bin Dou	Bian dou zi, Xiao bian dou	Herb	Stem, leaf	0	0	0	0	0	1	1	0.32
Leguminosae	*Lespedeza davurica* (Laxm.) Schindl.	Xin An Hu Zhi Zi	Hu shi tiao	Shrub	Flower, fruit	0	6	55	47	0	34	142	45.08
Leguminosae	*Lespedeza potaninii* Vassilcz.	Niu Zhi Zi	Hu shi tiao	Shrub	Flower, fruit	0	6	56	46	0	34	142	45.08
Leguminosae	*Medicago falcata* L.	Ye Mu Xu	Huang hua ye mu xu	Herb	Stem, leaf	6	0	45	47	38	40	176	55.87
Leguminosae	*Medicago lupulina* L.	Tian Lan Mu Xu	Di mu xu	Herb	Stem, leaf	0	0	0	0	38	0	38	12.06
Leguminosae	*Medicago ruthenica* (L.) Trautv.	Hua Mu Xu	Ye mu xu, Qiao pi cao, Di mu xu	Herb	Stem, leaf	0	0	0	0	38	40	78	24.76
Leguminosae	*Medicago ruthenica* (L.)Ledeb.	Zi Hua Mu Xu	Zi hua mu xu	Herb	Stem, leaf	29	16	44	49	36	40	214	67.94
Leguminosae	*Melilotus albus* Medik.	Bai Hua Cao Mu Xi	Ma mu xu	Herb	Stem, leaf	0	0	0	0	0	39	39	12.38
Leguminosae	*Melilotus officinalis* (L.) Pall.	Huang Hua Cao Mu Xi	Ma mu xu	Herb	Stem, leaf	0	0	0	0	0	39	39	12.38
Leguminosae	*Onobrychis viciifolia* Scop.	Lv Shi Cao	Hong dou cao	Herb	Stem, leaf	2	0	0	0	36	0	38	12.06
Leguminosae	*Oxytropis aciphylla* Ledeb.	Ci Ye Bing Ji Dou	Mao tou chai, Xiao mao tou	Shrub	Stem, leaf, flower	0	0	60	44	0	27	131	41.59
Leguminosae	*Pisum sativum* L.	Wan Dou	Wan dou	Herb	Stem, leaf, fruit	0	0	0	0	4	0	4	1.27
Leguminosae	*Robinia pseudoacacia* L.	Ci Huai	Yang huai, Huai shu	Tree	Stem, leaf	14	0	15	0	1	0	30	9.52
Leguminosae	*Sophora alopecuroides* L.	Ku Dou Zi	Ku du zi, Ye hu wan dou	Shrub	Fruit	0	0	3	0	0	30	33	10.48
Leguminosae	*Styphnolobium japonicum* (L.) Schott	Huai	Huai shu	Tree	Stem, leaf	0	0	15	0	1	0	16	5.08
Leguminosae	*Trifolium repens* L.	Bai Che Zhou Cao	Bai hua ye mu xu	Herb	Stem, leaf	0	0	34	8	38	0	80	25.4
Leguminosae	*Vicia amoena* Fisch.	Shan Ye Wan Dou	Ye wan dou	Herb	Stem, leaf	0	0	0	0	29	0	29	9.21
Leguminosae	*Vicia cracca* L.	Guang Bu Ye Wan Dou	Luo dou yang, Ye wan dou	Herb	Stem, leaf	0	0	3	1	29	1	34	10.79
Leguminosae	*Vicia faba* L.	Can Dou	Da dou	Herb	Stem, leaf	8	0	3	7	13	1	32	10.16
Leguminosae	*Vicia sepium* L.	Ye Wan Dou	Ye wan dou	Herb	Stem, leaf	0	0	0	0	29	0	29	9.21
Leguminosae	*Vicia unijuga* A.Br.	Wai Tou Cai	Wai tou cai	Herb	Stem, leaf	0	0	0	0	10	0	10	3.17
Rosaceae	*Potentilla anserina* L.	E Rong Wei Ling Cai	Jue ma	Herb	Root	0	0	0	0	4	0	4	1.27
Rosaceae	*Sibbaldianthe bifurca* (L.) Kurtto & T.Erikss.	Er Lie Wei Ling Cai	Tie pian zi, Hei gen zi yang, Ji guan cao	Herb or Shrub	Stem, leaf	0	0	53	26	5	35	119	37.78
Rosaceae	*Crataegus pinnatifida* Bunge	Shan Zha	Shan cha shu	Tree	Leaf	0	0	0	0	2	0	2	0.63
Rosaceae	*Duchesnea indica* (Jacks.) Focke	She Mei	Pie er, Mei zi	Herb	Stem, leaf	0	0	0	0	2	0	2	0.63
Rosaceae	*Fragaria orientalis* Losinsk.	Dong Fang Cao Mei	Ye cao mei, Pie er	Herb	Stem, leaf	0	0	0	0	2	0	2	0.63
Rosaceae	*Potentilla acaulis* L.	Xing Mao Wei Ling Cai	Ma shi shi	Herb	Leaf	0	0	0	0	0	12	12	3.81
Rosaceae	*Potentilla reptans* L.	Pu Fu Wei Ling Cai	Hong bang chui	Herb	Leaf	0	0	0	0	14	0	14	4.44
Rosaceae	*Prinsepia uniflora* Batalin	Rui He	Ma ru ci, Ma ru zi	Shrub	Leaf	0	0	53	36	0	0	89	28.25
Rosaceae	*Prunus davidiana* (CarriŠre) Franch.	Shan Tao	Shan mao tao, Ye tao	Shrub or Tree	Stem, leaf	0	0	3	26	1	21	51	16.19
Rosaceae	*Prunus sibirica* L.	Shan Xing	Xing zi, Heng zi	Shrub or Tree	Stem, leaf	0	0	11	26	1	23	61	19.37
Rosaceae	*Prunus tomentosa* Thunb.	Mao Ying Tao	Shan yin tao, Yin tao	Shrub	Leaf	0	0	0	0	1	0	1	0.32
Rosaceae	*Rubus parvifolius* L.	Mao Mei	Mei dou wan	Shrub	Leaf	0	0	0	0	8	0	8	2.54
Rosaceae	*Rubus parvifolius* var. taquetii (H. Lév.) Lauener & D.K. Ferguson	Xian Hua Mao Mei	Mei dou wan	Herb or Shrub	Leaf	0	0	0	0	8	0	8	2.54
Rosaceae	*Rubus pungens* Cambess.	Zhen Ci Xuan Gou Zi	Hei mei dou	Shrub	Stem, leaf	0	0	0	0	4	0	4	1.27
Rosaceae	*Spiraea pubescens* Turcz.	Tu Zhuang Xiu Xian Ju	Gan you ben zi	Shrub	Leaf	0	0	0	0	8	0	8	2.54
Elaeagnaceae	*Elaeagnus angustifolia* L.	Sha Zao	Sha zao zi	Shrub or Tree	Stem, leaf	0	0	3	27	0	0	30	9.52
Elaeagnaceae	*Elaeagnus rhamnoides* (L.) A.Nelson	Sha Ji	Sha bing, Hei ci	Shrub	Stem, leaf	0	0	32	31	12	1	76	24.13
Rhamnaceae	*Ziziphus jujuba* var. spinosa (Bunge) Hu ex H.F.Chow	Suan Zao	Suan zao zi, Shan zao zi	Shrub or Tree	Stem, leaf	0	0	3	33	0	0	36	11.43
Ulmaceae	*Ulmus pumila* L.	Yu Shu	Yu shu	Tree	Leaf	8	0	29	20	1	0	58	18.41
Moraceae	*Morus alba* L.	Sang	Sang shu	Shrub or Tree	Stem, leaf	0	0	0	0	1	0	1	0.32
Cucurbitaceae	*Citrullus lanatus* (Thunb.) Matsum. & Nakai	Xi Gua	Xi gua yang	Liana	Stem, leaf	0	0	1	44	0	0	45	14.29
Violaceae	*Viola philippica* Cav.	Zi Hua Di Ding	Dao jian yao	Herb	Stem, leaf	0	0	0	0	6	0	6	1.9
Linaceae	*Linum pallescens* Bunge	Duan Zhu Ya Ma	Ye hu ma	Herb	Flower, fruit	0	0	50	39	0	0	89	28.25
Linaceae	*Linum perenne* L.	Su Gen Ya Ma	Ye hu ma	Herb	Flower, fruit	0	0	0	0	0	11	11	3.49
Linaceae	*Linum stelleroides* Planch.	Ye Ya Ma	Ye hu ma	Herb	Stem, leaf, flower, fruit	0	0	50	39	1	0	90	28.57
Linaceae	*Linum usitatissimum* L.	Ya Ma	Jing zi hu ma	Herb	Stem, leaf, flower, fruit	0	0	16	10	1	13	40	12.7
Geraniaceae	*Erodium stephanianum* Willd.	Mang Niu Er Miao	Hong gen zi	Herb	Stem, leaf, flower	0	0	39	10	0	33	82	26.03
Geraniaceae	*Geranium sibiricum* L.	Shu Zhang Lao Guan Cao	Lao guan cao	Herb	Stem, leaf, flower	0	0	0	0	6	0	6	1.9
Nitrariaceae	*Nitraria tangutorum* Bobrov	Bai Ci	bia ci, Suan liu zi, Gai lia zi, Ga la mu	Shrub	Stem, leaf	0	0	2	19	0	24	45	14.29
Nitrariaceae	*Peganum harmala* L.	Luo Tuo Peng	Luo tuo peng, Luo luo peng	Herb	Stem, leaf	0	10	59	45	0	35	149	47.3
Nitrariaceae	*Peganum multisectum* (Maxim.) Bobrov	Duo Lie Luo Tuo Peng	Luo tuo peng, Luo luo peng	Herb	Stem, leaf	0	10	59	45	0	35	149	47.3
Nitrariaceae	*Peganum nigellastrum* Bunge	Luo Tuo Hao	Xiao luo tuo peng	Herb	Stem, leaf	0	0	59	45	0	0	104	33.02
Rutaceae	*Haplophyllum dauricum* (L.) G. Don	Bei Yun Xiang	Huang hua hua	Herb	Stem, leaf, flower	0	0	50	39	0	0	89	28.25
Malvaceae	*Alcea rosea* L.	Shu Kui	Ye wei hua	Herb	Stem, leaf, flower	0	0	0	0	12	0	12	3.81
Malvaceae	*Hibiscus trionum* L.	Ye Xi Gua Miao	Hei zi zi	Herb	Stem, leaf	0	0	0	0	0	6	6	1.9
Malvaceae	*Malva verticillata* L.	Dong Kui	Qi ye zi, Ye jing kui	Herb	Stem, leaf	3	0	0	0	2	26	31	9.84
Brassicaceae	*Braya humilis* (C.A. Mey.) B.L. Rob.	Yin Guo Jie	Bai hua zi, Que er nao nao	Herb	Stem, leaf	0	0	47	33	0	33	113	35.87
Brassicaceae	*Capsella bursa-pastoris* (L.) Medik.	Ji	Hua hua cai, Ji cai	Herb	Stem, leaf	0	0	0	0	1	0	1	0.32
Brassicaceae	*Descurainia sophia* (L.) Webb ex Prantl	Bo Niang Hao	Ye cai zi	Herb	Stem, leaf	0	0	0	0	18	0	18	5.71
Brassicaceae	*Draba nemorosa* L.	Ting Li	Niu ji jiao	Herb	Stem, leaf	0	0	0	0	21	0	21	6.67
Brassicaceae	*Eruca vesicaria* (L.) Cav.	Zhi Ma Cai	Yun gai, Yuan yuan	Herb	Seed	0	0	21	8	9	27	65	20.63
Brassicaceae	*Lepidium apetalum* Willd.	Du Xing Cai	La la yang, La la ying	Herb	Stem, leaf	0	11	47	26	7	22	113	35.87
Brassicaceae	*Lepidium latifolium* L.	Kuan Ye Du Xing Cai	Da la la	Herb	Stem, leaf	0	0	0	0	0	14	14	4.44
Brassicaceae	*Malcolmia africana* (L.) R.Br.	Se Ji	Tian luo bo	Herb	Stem, leaf	0	0	0	0	0	20	20	6.35
Brassicaceae	*Thlaspi arvense* L.	Xi Mi	Ku gai zi	Herb	Stem, leaf	0	0	0	0	1	0	1	0.32
Tamaricaceae	*Reaumuria soongarica*	Hong Sha	Hong xiang chai, Hong xun chai	Shrub	Stem, leaf	0	0	48	38	0	0	86	27.3
Tamaricaceae	*Tamarix chinensis* Lour.	Guai Liu	Guai liu, Hong liu	Shrub or Tree	Stem, leaf, flower	0	0	4	9	0	0	13	4.13
Plumbaginaceae	*Limonium bicolor* (Bunge) Kuntze	Er Se Bu Xue Cao	Xiao hua hua, Ma niu niu	Herb	Leaf, flower	0	0	29	20	0	22	71	22.54
Polygonaceae	*Calligonum mongolicum* Turcz.	Sha Guai Zao	Sha zao zi	Shrub	Stem, leaf, fruit	0	0	9	43	0	0	52	16.51
Polygonaceae	*Fagopyrum esculentum* Moench	Qiao Mai	Qiao mai, Tian qiao	Herb	Stem, leaf	6	0	4	3	1	1	15	4.76
Polygonaceae	*Fagopyrum tataricum* (L.) Gaertn.	Ku Qiao	Ku qiao	Herb	Stem, leaf	0	0	14	3	1	1	19	6.03
Polygonaceae	*Persicaria lapathifolia* (L.) Delarbre	Suan Mo Ye Liao	Da ye suan bu liu liu, Da ma liao	Herb	Stem, leaf	0	0	0	0	15	0	15	4.76
Polygonaceae	*Persicaria vivipara* (L.) Ronse Decr.	Zhu Ya Liao	Tie xiu xiu, Hong san qi, Qiao mai qi	Herb	Stem, leaf	0	0	51	43	1	0	95	30.16
Polygonaceae	*Polygonum aviculare* L.	Bian Xu	Bian xu zi, Ye sao zhou	Herb	Stem, leaf	0	0	51	43	2	10	106	33.65
Polygonaceae	*Polygonum sibiricum* Laxm.	Xi Bo Li Ya Liao	Suan liu liu, Mian tiao	Herb	Stem, leaf, flower	0	0	7	43	0	10	60	19.05
Polygonaceae	*Rheum palmatum* L.	Zhang Ye Da Huang	Dai huang, Zhang ye da huang	Herb	Stem, leaf	0	0	51	49	14	16	130	41.27
Polygonaceae	*Rheum tanguticum* Maxim. ex Balf.	Ji Zhua Da Huang	Dai huang, Liu pan shan ji zhau da huang	Herb	Stem, leaf	0	0	0	0	14	0	14	4.44
Polygonaceae	*Rumex acetosa* L.	Suan Mo	Xiao ye suan bu liu liu, Xiao ye suan mo	Herb	Stem, leaf	0	0	0	0	14	0	14	4.44
Polygonaceae	*Rumex crispus* L.	Zhou Ye Suan Mo	Lv er duo, Tu dai huang	Herb	Stem, leaf	0	0	51	49	4	2	106	33.65
Polygonaceae	*Rumex patientia* L.	Ba Tian Suan Mo	Lv er duo guang zi, Lv er gua	Herb	Stem, leaf	0	0	51	49	4	2	106	33.65
Caryophyllaceae	*Dianthus chinensis* L.	Shi Zhu	Hong chou zi hua	Herb	Stem, leaf	0	0	0	0	22	0	22	6.98
Caryophyllaceae	*Dianthus superbus* L.	Qu Mai	Qu mai	Herb	Stem, leaf	0	0	0	0	5	0	5	1.59
Caryophyllaceae	*Stellaria dichotoma* var. lanceolata Bunge	Yin Chai Hu	Yin chai hu	Herb	Stem, leaf	0	0	0	0	11	0	11	3.49
Amaranthaceae	*Salsola kali* subsp. tragus (L.) Čelak.	Ci Sha Peng	Ci peng	Herb	Stem, leaf	30	21	29	30	6	35	151	47.94
Amaranthaceae	*Salsola collina* Pall.	Zhu Mao Cai	Ci peng, Zheng yan zi zha li zi, Peng zi cai	Herb	Stem, leaf	32	18	67	50	23	35	225	71.43
Amaranthaceae	*Salsola passerina* Bunge	Zhen Zhu Zhu Mao Cai	Zhen zhu chai, Ha ma tou	Shrub	Stem, leaf	0	0	60	49	0	0	109	34.6
Amaranthaceae	*Agriophyllum squarrosum* (L.) Moq.	Sha Peng	Deng suo	Herb	Stem, leaf, seed	0	0	60	49	0	0	109	34.6
Amaranthaceae	*Amaranthus retroflexus* L.	Fan Zhi Xian	Ye ren han, Gan sui gu	Herb	Stem, leaf, flower, seed	0	0	1	0	32	0	33	10.48
Amaranthaceae	*Atriplex centralasiatica* Iljin	Zhong Ya Bin Li	Yang er duo hui tiao, Ma luo luo	Herb	Stem, leaf	0	0	0	0	0	37	37	11.75
Amaranthaceae	*Atriplex fera* (L.) Bunge	Ye Bin Li	Yang er duo hui tiao	Herb	Stem, leaf	0	0	0	0	0	36	36	11.43
Amaranthaceae	*Atriplex sibirica* L.	Xi Bo Li Ya Bin Li	Ma hui tiao	Herb	Stem, leaf	0	0	62	56	0	0	118	37.46
Amaranthaceae	*Chenopodium album* L.	Li	Hui tiao	Herb	Stem, leaf	18	12	62	57	33	36	218	69.21
Amaranthaceae	*Chenopodium glaucum* L.	Hui Lv Li	Hui tiao	Herb	Stem, leaf	17	0	62	57	33	36	205	65.08
Amaranthaceae	*Chenopodium hybridum* L.	Za Pei Li	Hui tiao	Herb	Stem, leaf	0	0	0	0	33	0	33	10.48
Amaranthaceae	*Dysphania schraderiana* (Schult.) Mosyakin & Clemants	Ju Ye Xiang Li	Xiao ye hui tiao	Herb	Stem, leaf	0	0	0	0	32	0	32	10.16
Amaranthaceae	*Halogeton arachnoideus*	Bai Jing Yan Sheng Cao	Shui peng hao, Shui hao	Herb	Stem, leaf	0	5	4	49	34	33	125	39.68
Amaranthaceae	*Haloxylon ammodendron* (C.A.Mey.) Bunge ex Fenzl	Suo Suo	Suo suo	Shrub or Tree	Stem, leaf	0	0	18	43	0	0	61	19.37
Amaranthaceae	*Kalidium cuspidatum* (Ung.-Sternb.) Grubov	Jian Ye Yan Zhua Zhua	Lao shu shi dan dan, Yan hao	Shrub	Stem, leaf	0	0	42	43	0	0	85	26.98
Amaranthaceae	*Krascheninnikovia ceratoides* (L.) Gueldenst.	Tuo Rong Li	You ruo li	Shrub	Stem, leaf, flower, fruit	0	0	29	43	0	0	72	22.86
Amaranthaceae	*Suaeda glauca* (Bunge) Bunge	Jian Peng	Lv wei ba yan hao, Yan hao hao, Jian hao, Jian peng	Herb	Stem, leaf	0	6	60	54	0	34	154	48.89
Amaranthaceae	*Sympegma regelii* Bunge	He Tou Cao	Hei chai, He chai, Hei ma tou chai, He lao gua chai	Shrub	Stem, leaf	0	0	42	43	0	0	85	26.98
Amaranthaceae	*Bassia scoparia* (L.) A.J.Scott	Di Fu	Mao luo li, Li jing, Ye li jing	Herb	Stem, leaf	11	0	42	35	1	11	100	31.75
Amaranthaceae	*Corispermum patelliforme*	Die Guo Chong Shi	Mian peng	Herb	Stem, leaf, seed	0	17	60	55	0	38	170	53.97
Amaranthaceae	*Corispermum patelliforme* Iljin	Sheng Chong Shi	Mian peng	Herb	Stem, leaf	0	17	61	54	0	38	170	53.97
Talinaceae	*Pedicularis muscicola* Maxim.	Xian Sheng Ma Xian Hao	Chang chong cao	Herb	Stem, leaf	0	0	0	0	1	0	1	0.32
Portulacaceae	*Portulaca oleracea* L.	Ma Chi Xian	Pang wa wa cai	Herb	Stem, leaf	1	5	12	9	19	2	48	15.24
Rubiaceae	Galium aparine L.	Zhu Yang Yang	Ran wa zi cao	Herb	Stem, leaf	0	0	0	0	19	0	19	6.03
Rubiaceae	*Galium verum* L.	Peng Zi Cai	Huang mi gan fan	Herb	Stem, leaf, Flower	0	0	0	0	1	0	1	0.32
Rubiaceae	*Rubia cordifolia* L.	Qian Cao	Qian cao zi, Ran wa zi	Liana	Stem, leaf	0	0	2	0	3	0	5	1.59
Apocynaceae	*Cynanchum absconditum* Liede	Di Shao Gua	Hao gua zi	Herb	Leaf, fruit	0	0	38	20	0	31	89	28.25
Apocynaceae	*Cynanchum acutum* L.	Yang Jiao Zi Cao	Yang nai jiao jiao, Yang jiao zi	Herb	Leaf	0	0	25	48	0	6	79	25.08
Apocynaceae	*Cynanchum chinense* R.Br.	E Rong Teng	Yang nai jiao jiao, Ma hao gua zi	Herb	Leaf	0	1	55	48	0	0	104	33.02
Boraginaceae	*Lappula myosotis* V. Wolf	He Shi	Ran ran zi, Mao ran ran	Herb	Stem, leaf	0	4	58	58	0	30	150	47.62
Boraginaceae	*Lappula squarrosa* (Retz.) Dumort.	Lan Ci He Shi	Zhan sheng cao	Herb	Stem, leaf	0	0	0	0	13	0	13	4.13
Boraginaceae	*Lappula squarrosa* subsp. heteracantha (Ledeb.) Chater	Yi Ci He Shi	Ran ran zi, Mao ran ran	Herb	Stem, leaf	0	6	64	58	0	30	158	50.16
Convolvulaceae	*Calystegia hederacea* Wall.	Da Wan Hua	Fu zi miao, Tian xuan hua, Gu zi man, Ku zi man, La ba hua, Da da wai, Bai hua gu zi man	Herb	Stem, leaf, flower	3	7	1	56	30	35	132	41.9
Convolvulaceae	*Convolvulus arvensis* L.	Tian Xuan Hua	Gu zi man, Ku zi man, Fen hua gu zi man	Herb	Stem, leaf, flower	3	7	55	39	2	35	141	44.76
Convolvulaceae	*Convolvulus tragacanthoides* Turcz.	Ci Xuan Hua	Ying zhua ci, Tie dan dan	Shrub	Stem, leaf, flower	0	0	62	39	0	0	101	32.06
Convolvulaceae	*Cuscuta chinensis* Lam.	Tu Si Zi	Huang tang, Huang chan	Herb	Stem, leaf	0	0	11	41	0	0	52	16.51
Convolvulaceae	*Ipomoea purpurea* (L.) Roth	Yuan Ye Qian Niu	Qian niu hua, La ba hua, Hei bai chou	Herb	Stem, leaf, flower	4	3	21	4	3	22	57	18.1
Solanaceae	*Lycium barbarum* L.	Ning Xia Gou Qi	Gou ji zi, Gou qi zi	Shrub	Stem, leaf	0	0	15	1	0	6	22	6.98
Solanaceae	*Lycium chinense* Mill.	Gou Qi	Gou ji zi, Gou qi zi	Shrub	Stem, leaf	0	0	15	1	0	6	22	6.98
Solanaceae	*Solanum americanum* Mill.	Long Kui	Ye wei hua, Ye hua hua	Herb	Stem, leaf	0	0	0	0	12	0	12	3.81
Solanaceae	*Solanum tuberosum* L.	Ma Ling Shu	Yang yu	Herb	Stem, leaf	0	0	0	0	0	4	4	1.27
Plantaginaceae	*Plantago asiatica* L.	Che Qian	Che qian cao, Niu she tou, Niu er duo	Herb	Stem, leaf, flower, fruit	7	2	0	0	28	14	51	16.19
Plantaginaceae	*Plantago depressa* Willd.	Ping Che Qian	Che qian cao, Niu she tou, Niu er duo	Herb	Stem, leaf	7	2	0	0	28	14	51	16.19
Plantaginaceae	*Plantago major* L.	Da Che Qian	Che qian cao, Niu she tou, Niu er duo	Herb	Stem, leaf, flower, fruit	7	2	0	0	28	14	51	16.19
Lamiaceae	*Mentha canadensis* L.	Bo He	Ye bo he	Herb	Stem, leaf	0	0	0	0	1	0	1	0.32
Lamiaceae	*Lagochilus ilicifolius* Bunge ex Benth.	Dong Qing Ye Tu Chun Hua	Ji guan zi	Herb	Stem, leaf	0	0	1	23	0	0	24	7.62
Lamiaceae	*Dracocephalum heterophyllum* Benth.	Bai Hua Zhi Zi Hua	Mi guan guan	Herb	Stem, leaf	0	0	0	0	1	10	11	3.49
Lamiaceae	*Leonurus japonicus* Houtt.	Yi Mu Cao	Jie jie hao	Herb	Stem, leaf	0	0	18	10	0	0	28	8.89
Lamiaceae	*Stachys affinis* Bunge	Gan Lu Zi	Di liu zi	Herb	Stem, leaf	0	2	6	2	1	0	11	3.49
Lamiaceae	*Thymus mongolicus* (Ronniger) Ronniger	Bai Li Xiang	Di jiao	Shrub	Stem, leaf	0	0	0	0	20	1	21	6.67
Campanulaceae	*Adenophora potaninii* Korsh.	Pao Sha Shen	Niu ling hua	Herb	Stem, leaf	0	0	0	0	17	0	17	5.4
Campanulaceae	*Codonopsis pilosula* (Franch.) Nannf.	Dang Shen	Dang sen	Herb	Stem, leaf	0	0	0	0	19	0	19	6.03
Compositae	*Ajania achilleoides* (Turcz.) Poljakov ex Grubov	Shi Zhuang Ya Ju	Ga ji hao, bia mi hao	Shrub	Leaf	0	0	29	42	0	0	71	22.54
Compositae	*Ajania fruticulosa* (Ledeb.) Poljakov	Guan Mu Ya Ju	Ga ji hao	Shrub	Leaf	0	0	29	32	0	0	61	19.37
Compositae	Arctium lappa L.	Niu Pang	Niu zi, Da li zi	Herb	Stem, leaf	0	0	0	0	1	0	1	0.32
Compositae	*Artemisia annua* L.	Huang Hua Hao	Chou hao, Huang hao	Herb	Stem, leaf	0	0	29	31	30	27	117	37.14
Compositae	*Artemisia argyi* H.Lév. & Vaniot	Ai	Nai, Ai	Herb	Flower	5	5	10	2	27	29	78	24.76
Compositae	*Artemisia blepharolepis* Bunge	Bai Sha Hao	bia sha hao	Shrub	Stem, leaf	4	5	51	54	0	27	141	44.76
Compositae	*Artemisia capillaris* Thunb.	Yin Chen Hao	Bai hao tou zi, You hao	Herb or Shrub	Stem, leaf	18	21	61	49	34	30	213	67.62
Compositae	*Artemisia desertorum* Spreng.	Hei Sha Hao	Sha hao, You hao	Shrub	Stem, leaf	4	16	52	55	0	27	154	48.89
Compositae	*Artemisia dubia* L. ex B.D.Jacks.	Wu Mao Niu Wei Hao	Yi zi hao	Herb	Leaf	0	0	51	0	0	0	51	16.19
Compositae	*Artemisia frigida* Willd.	Len Hao	Chuan di hao	Herb	Stem, leaf	0	0	29	31	0	0	60	19.05
Compositae	*Artemisia giraldii* Pamp.	Hua Bei Mi Hao	Jiao hao	Herb	Stem, leaf, fruit	0	0	51	38	0	0	89	28.25
Compositae	*Artemisia gmelinii* Weber ex Stechm.	Bai Lian Hao	A ji hao, Dai mi hao, Ying gan gan hao, Tie gan hao	Herb	Stem, leaf	0	0	29	31	18	24	102	32.38
Compositae	*Artemisia scoparia* Waldst. & Kitam.	Zhu Mao Hao	Bai hao tou zi , You hao	Herb	Stem, leaf	18	21	62	59	34	36	230	73.02
Compositae	*Carduus crispus* Guirão ex Nyman	Si Mao Fei Lian	Ci gai	Herb	Leaf, flower	0	4	0	0	0	25	29	9.21
Compositae	*Cirsium arvense* (L.) Scop.	Ci Er Cai	Ma ci ji, Ci ji gai, Xiao ji, Da ji	Herb	Leaf, flower	0	4	25	18	0	25	72	22.86
Compositae	*Ixeris chinensis* (Thunb. ex Thunb.) Nakai	Zhong Hua Xiao Ku Mai	Yan ji ji cao, Gua la ji cao, Ma yan wo cao	Herb	Stem, leaf	0	8	53	45	0	28	134	42.54
Compositae	*Helianthus annuus* L.	Xiang Ri Kui	Xiang ri kui	Herb	Stem, leaf	11	5	35	36	7	30	124	39.37
Compositae	*Helianthus tuberosus* L.	Ju Yu	Yang jiang	Herb	Stem, leaf, flower	0	0	52	2	0	0	54	17.14
Compositae	*Heteropappus altaicus* (Willd.) Novopokr.	A Er Tai Gou Wa Hua	Bai hua cao	Herb	Stem, leaf, flower	0	0	29	33	0	28	90	28.57
Compositae	*Lactuca tatarica* (L.) C.A.Mey.	Ru Ju	Ma ku ku cai	Herb	Stem, leaf	12	0	54	55	0	45	166	52.7
Compositae	*Neopallasia pectinata* (Pall.) Poljakov	Zhi Ye Hao	Mi hao, Mei hao	Herb	Stem, leaf	0	0	29	43	0	22	94	29.84
Compositae	*Rhaponticum repens* (L.) Hidalgo	Ding Yu Ju	Ku hao	Herb	Leaf, flower	0	3	18	29	0	33	83	26.35
Compositae	*Saussurea alata* DC.	Yi Jing Feng Mao Ju	Ye da dou	Herb	Stem, leaf	0	0	0	0	12	0	12	3.81
Compositae	*Scorzonera divaricata* Turcz.	Cha Zhi Ya Cong	Nai gua zi	Herb	Stem, leaf	0	19	10	33	0	31	93	29.52
Compositae	*Sonchus arvensis* L.	Ju Mai Cai	Ku ku cai, Ku xu, Tian ku ku cai	Herb	Stem, leaf	17	6	54	55	0	45	177	56.19
Compositae	*Sonchus oleraceus* (L.) L.	Ku Ju Cai	Ku ku cai, Tian ku cai	Herb	Stem, leaf	17	6	54	55	0	45	177	56.19
Compositae	*Taraxacum mongolicum* Hand.-Mazz.	Pu Gong Ying	Huang huang zi, Huan huan tai	Herb	Stem, leaf	18	4	54	0	0	39	115	36.51
Compositae	*Tussilago farfara* L.	Kuan Dong	Dong hua	Herb	Stem, leaf	0	0	0	0	1	0	1	0.32
Compositae	*Xanthium strumarium* subsp. sibiricum (Patrin ex Widder) Greuter	Cang Er	Cang er zi, Cao er	Herb	Stem, leaf	0	2	13	20	0	21	56	17.78
Apiaceae	*Ligusticum striatum* DC.	Chuan Xiong	Chuan xiong	Herb	Stem, leaf	0	0	0	0	1	0	1	0.32
Apiaceae	*Anthriscus sylvestris* (L.) Hoffm.	E Shen	Ye hong luo bo	Herb	Stem, leaf	0	0	0	0	15	0	15	4.76
Apiaceae	*Bupleurum chinense* DC.	Bei Chai Hu	Da er duo chai hu	Herb	Stem, leaf	0	0	0	0	18	0	18	5.71
Apiaceae	*Daucus carota* L.	Hu Luo Bo	Hu luo bo	Herb	Root	0	0	25	12	0	0	37	11.75
Apiaceae	*Ferula bungeana* Kitag.	Sha Hui Xiang	Mian diao diao	Herb	Leaf	0	0	0	0	0	4	4	1.27
Apiaceae	*Ligusticum sinense* Oliv.	Gao Ben	Ye chuan xiong	Herb	Stem, leaf	0	0	0	0	3	0	3	0.95
Apiaceae	*Saposhnikovia divaricata* (Turcz.) Schischk.	Fang Feng	Han sa jiao, Ma yin zi	Herb	Stem, leaf	0	0	0	0	6	0	6	1.9
Apiaceae	*Torilis japonica* (Houtt.) DC.	Xiao Qie Yi	Ye hong luo bo, Ye hui xiang	Herb	Leaf	0	0	0	0	34	0	34	10.79
Ephedraceae	*Ephedra intermedia* Schrenk & C.A.Mey.	Zhong Ma Huang	Ma huang	Shrub	Stem, leaf	0	0	51	49	0	17	117	37.14
Equisetaceae	*Equisetum arvense* L.	Wen Jing	Tuan xu, Duan xu	Herb	Leaf	0	0	0	0	6	0	6	1.9
Equisetaceae	*Equisetum ramosissimum* Desf.	Jie Jie Cao	Jie jie cao, Jie jie tiao	Herb	Leaf	0	0	0	0	6	0	6	1.9

### Value and knowledge of major forage plants for feeding livestock

The RFC value calculated in this study ranged from 0.32 to 77.14. For each forage plant species, the higher the RFC value, the more frequently it is used by local villagers to feed livestock, and the more important and valuable it is in the area. The top 13 plant species with an RFC value greater than 50 were *Agropyron cristatum* (RFC 77.1), *Artemisia scoparia* (RFC 73.0), *Salsola collina* (RFC 71.4), *Chenopodium album* (RFC 69.2), *Medicago ruthenica* (RFC 67.9), *Artemisia capillaris* (RFC 67.6), *Setaria viridis* (RFC 67.3), *Chenopodium glaucum* (RFC 65.1), *Zea mays* (RFC 56.8), *Sonchus* (RFC 56.2), *Sonchus oleraceus* (RFC 56.2), *Medicago falcata* (RFC 55.9), and *Echinochloa crus-galli* (RFC 54.9). These plants have a good palatability and are favorite feed of cattle and sheep. Their stems and leaves are the common parts used for livestock feed in the form of either tender grass or green hay. Among them, *maize* (*Z. mays*) and *alfalfa* (*M. ruthenica*) are cultivated forage grasses with high forage value. *Wheatgrass* (*A. cristatum*), *green bristlegrass* (*S. viridis*), and *paspalum* (*E. crus-galli*) have a massive amount of leaves and soft stems favored by cattle and sheep. *M*. *falcata* is favored by domestic livestock during the entire plant growth period and after withering; green and tender plants can increase the milk yield of dairy livestock, stems left after defoliation in winter are favorite forage of livestock, and the green hay of this species is also good winter forage. For *C. glaucum*, despite its high RFC value as forage for both cattle and sheep in the forms of either green tender plants or dry hay, the villagers warned that the plants can cause itching or swelling of the whole body if livestock consumes too much.

*S. arvensis*, *S. oleraceus*, and *L. tatarica* are three important local forage plants with RFC values of 56.2, 56.2, and 52.7, respectively. These three forage plant species, which were mentioned by all the respondents from Village f, share a common Chinese name, *Kukucai*, because of their bitter taste. They are edible and medicinal and can be used for livestock feed. *Kukucai* is commonly used in a homemade local cold dish. When used as an anti-inflammatory medicine, it is commonly referred to as *Patrinia villosa*. In addition, the juicy stems and leaves of *Kukucai* are favorite forage of cattle and sheep. These applications fully reflect the important value of *Kukucai*.

### Comparison of traditional forage plants in six villages in the study area

Among the 224 identified plants, 40 species (18%) were mentioned in Village a, 52 species (23%) in Village b, 132 species (59%) in Village c, 123 species (55%) in Village d, 121 species (54%) in Village e, and 118 species (53%) in Village f. In terms of the number of forage plant species reported, Village c > Village d > Village e > Village f > Village b > Village a. Villagers in Villages a and b reported fewer forage plant species, only approximately 20% of the total. In contrast, Villages c, d, e, and f each mentioned more than 100 forage plant species, approximately 50% of the total forage plant species reported. The number of forage plant species used by the villagers in Villages a and b was significantly lower, by 33% and 44%, respectively, than the number of those used by the villagers in Villages e and f near their original living areas. Long-distance migration brought prominent changes in the ecological environment and natural resources to the villagers of Villages a and b. In particular, for Village a, which moved from a forest area to a semiarid desertified area, the number of traditional forage plant species used by villagers showed a significant decreasing trend, indicating that the relocation had a great impact on the diversity of traditional knowledge regarding forage plants. Contrarily, Villages c and d, whose inhabitants migrated less than 2 km, experienced little change in the ecological environment and natural resources, and the short-distance relocation had a nonsignificant impact on the diversity of and knowledge regarding forage plants, as evidenced by the relatively small change in the number of traditional forage plant species used by villagers.

**Fig. 3:**
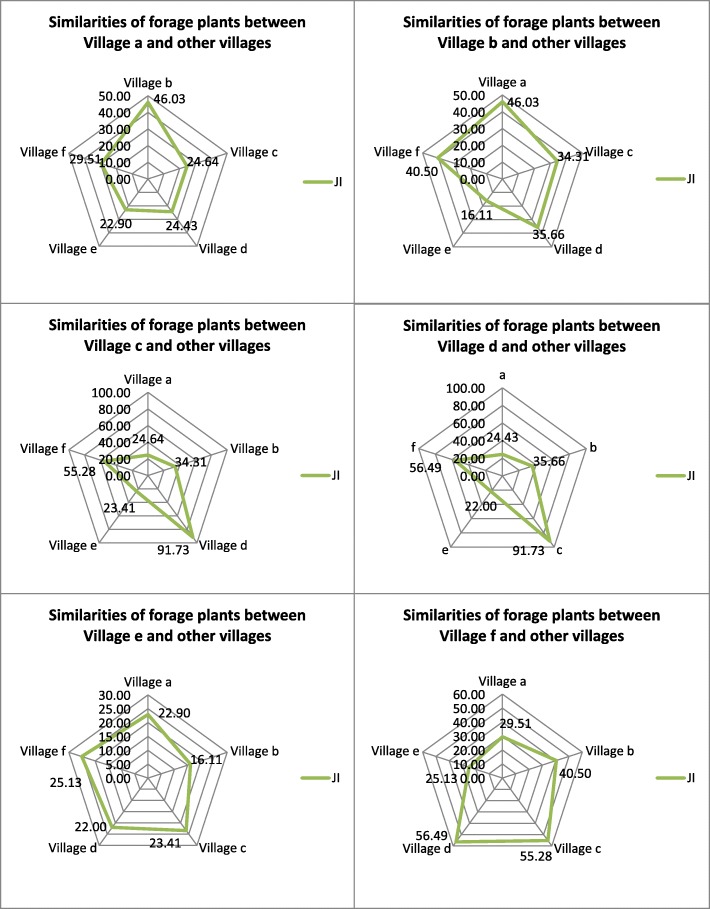
Similarities in traditional forage plants between six villages

In this study, the JI was used to represent the similarities in traditional forage plants among the six villages (Fig. 3). The higher the JI is, the greater the similarity in forage plant utilization between two villages. Village a has a similarity of 46.03 to Village b, higher than that to Village e nearby its emigration location, indicating that the utilization of forage plants by Village a is highly similar to the village nearby its immigration location but largely different from its emigration location. Village b shows a high similarity to Village a (46.03) and to Village f (40.50), indicating that the villagers in Villages b adapted themselves to the new environment through knowledge exchange and fusion with the residents in the immigration area while maintaining a certain homology with Village f, from where they moved. This might be attributed to the similarity in plant species between the loess hilly area from where Village b originated and its current location in the semiarid desertified area. In addition to a similarity in the number of forage plant species, Villages c and d show a high similarity (91.73) in the types of forage plant species, indicating that the two villages are highly similar in the use of forage plants because of a close historiogeographical relationship and similar ecological environment and natural resources.

**Fig. 4:**
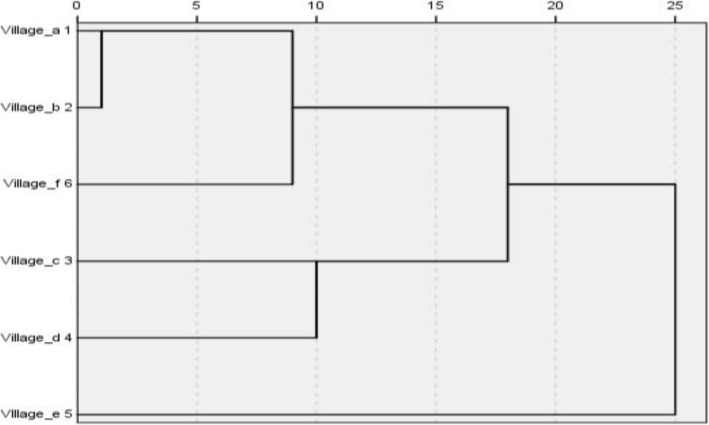
Tree diagram of a cluster analysis of similarities in forage plant species utilization among six villages

Figure 4 presents a cluster tree for the similarity of forage plant species among the six villages. In the first cluster, Village b shows the closest relationship with Village a, followed by its relationship with Village f, fully proving that there are certain homologies in the utilization of forage plants between the immigration and emigration areas. Village a has low similarity to and a large distance function with respect to Village e, near where it originated, which might be related to the large difference in natural resources between the two villages. In the second cluster, Villages c and d show the closest relationship in terms of forage plant utilization, suggesting that the two villages have high similarity in forage plant diversity and related knowledge after a short-distance migration within the same ecological type. This finding is consistent with the results from the JI analysis between the villages. Our findings were consistent among different analysis methods.

### Effects of gender, age, education level, and occupation on traditional forage plant knowledge

The distribution of traditional forage plant knowledge among local populations varies with regard to gender, age, education level, and occupation. As shown in Tables 5 and 6, knowledge level is significantly correlated with gender (*P* < 0.05), age (*P* < 0.05), education level (*P* < 0.05), and occupation (*P* < 0.05). Among 315 people interviewed in this survey, there were 165 males and 150 females, accounting for 52% and 48% of the total, respectively. Males have more knowledge of traditional forage plants than do females (*P* = 0.005, pairwise comparison). The mean number of traditional forage plant species reported increased by age group: 15.196 for the respondents under 19 years old, 42.472 for respondents 20–39 years old, 49.826 for the respondents 40–59 years old, and 51.352 for the respondents over 60 years old. The respondents over 40 years old know the most about traditional forage plant species, but there is no significant difference between 40–59 years old respondents and respondents over 60 years old regarding the mean number of plant species reported (*P* = 0.660, pairwise comparison). The respondents between 20 and 39 years old know less, and respondents under 19 years old know the least. From the perspective of education level, the uneducated population (illiterate) knows the most about traditional forage plant species (mean number 52.440) and have the most abundant knowledge (*P* value ≤ 0.05, pairwise comparison of this population to people with an education level of primary school, middle school, high school, technical secondary school, or above) in terms of the number of forage plant species reported. From the perspective of occupation, migrant workers (mean 37.488) have relatively less knowledge of traditional forage plants, while students (mean 17.417) have the least knowledge (*P* value < 0.001, pairwise comparison of students to people of other occupations). In summary, males over the age of 40 and with a low education level have more understanding and a rich knowledge of traditional forage plants, while students and those under 19 years old have less knowledge regarding traditional forage plants and livestock feeding.

**Table 5:** Knowledge of forage plants based on the characteristics of respondents from five villages

Characters	Total number of respondents	Average kinds of the forage plants mentioned		*p* value
Gender			4844.385, df = 1	0.000
Male	165	45.153		
Female	150	37.612		
Age range			10789.559, df = 3	0.000
≤ 19	28	15.196		
20–39	85	42.472		
40–59	127	49.826		
≥ 60	75	51.352		
Education Level			1758.473, df = 3	0.002
Illiteracy	128	52.440		
Primary	119	39.951		
Middle	49	42.831		
Higher	19	22.529		
Occupation			984.535, df = 6	0.013
Village heads	12	64.750		
Farmers	244	43.630		
businessmen	5	52.600		
Migrant workers	19	37.488		
Village doctors	5	45.800		
Students	23	17.417		
Other else	7	47.571

**Table 6:** Pairwise comparisons of the number of traditional forage plant species reported

Characters	Mean difference	S.t.d. error	*p* value
Gender (i-j)			
“Male”–“Female”	7.541	2.647	0.005
Age (i-j)			
“≥60”–“≤19”	36.156	4.580	0.000
“≥60”–“20–39”	8.880	3.559	0.014
“≥60”–“40–59”	1.526	3.463	0.660
Education level (i-j)			
“Illiteracy”–“Primary”	12.490	3.032	0.000
“Illiteracy”–“Middle”	9.609	3.673	0.010
“Illiteracy”–“Higher”	29.911	4.981	0.000
Occupation (i-j)			
“Students”–“Village heads”	− 47.333	6.829	0.000
“Students”–“Farmers”	− 26.213	4.413	0.000
“Students”–“businessmen”	− 35.183	9.369	0.000
“Students”–“Migrant workers”	− 20.071	6.256	0.002
“Students”–“Village doctors”	− 28.383	9.369	0.003
“Students”–“Other else”	− 30.155	8.223	0.000

## Discussion

Several years after relocation, new natural and human environments that differ from the place of emigration can gradually change the utilization behavior and the knowledge of ecological migrants towards plant resources [24, 35]. The diversity of traditional forage plant resources and related knowledge retained by ecological immigrants may significantly change after migration. Even though ecological migrants still apply traditional knowledge in their livelihood after migration, knowledge inheritance becomes a concern. The diversity of traditional forage plant resources used by immigrants refers to the types, quantity, and parts of traditional forage plants that can be used as forage [2, 9, 36], which depends, to a certain extent, on the distribution of forage plant resources around the area they live. The related knowledge includes the knowledge about forage forms of, seasonal use of, suitable livestock for traditional forage plants, and classification of pasture habitats [1, 5, 8, 37–40], which is affected by certain cultural factors, such as livestock rearing and breeding habits, as well as traditional techniques. Previous studies have concluded that the factors affecting traditional plant resources and associated knowledge include the natural environment (ecosystem [41]), traditional culture [42], customs and habits [43], the theoretical system of traditional food and medicine [10, 44], traditional production and lifestyle [45, 46], and degree of modernization [47, 48]. The present study found that the diversity of traditional forage plants shows both differences and similarities between immigration and emigration locations and that the degree of preservation is closely related to the ecological environment, traditional culture, customs and habits, production and lifestyle [49–51].

Forage plant resources in Ningxia have a diverse distribution due to Ningxia’s complex geomorphological structure: the Liupan Mountain water conservation forest in the south, Loess Plateau and Jianshan Basin in the mid-south area, alluvial plain of the Yellow River in the mid-north, and semiarid desertified area partially covering central Ningxia. Diverse ecological environments have resulted in diverse vegetation types and plant species in Ningxia. Throughout history, Ningxia has been a mixed agricultural-pastoral area. Immigration villages a, b, c, and d in Hongsibu District of Ningxia are in a desert steppe desertification control area in central Ningxia. Therefore, the forage plants used by the villagers have psammophyte, xerophyte, and halophyte characteristics. Village f is located near the emigration area in a loess hilly region where the vegetation possesses typical xerophyte and halophyte characteristics. Village e is located near the emigration area in a forest where the vegetation possesses typical damp (shady) and semidamp (semishady) mountain plant characteristics. In this study, the immigrants in four villages all reported more than 100 forage plant types, but the vegetation types vary among villages. The villagers in Villages c, d, and f are good at raising sheep; hence, they listed a variety of psammophytic, xerophytic, and halophytic plants and knew many forage plants for sheep in semiarid areas. Moreover, the plants reported by Villages c and d are highly similar but very different from those reported by Village f, indicating a homologous relationship between immigrants from Villages c and d. The villagers in Village e are good at raising cattle, and their “Jingyuan Yellow Cattle” is a well-known local geographical indication product. The local forage plant species reported by the villagers are highly diverse, and the forage plant knowledge of the villagers is mostly related to the plants that cattle like to eat. This can be attributed to the unique forest landscape and plant resources in the area as well as to traditional cattle-raising experience and knowledge. In conclusion, the diversity of traditional forage plants reported is related to the regional distribution of plant resources. However, we also found a concerning problem. Villages a and b, who migrated from a forest area and loess hilly area to a semiarid desertified area, experienced prominent changes in the natural environment and forage plant resources. The villagers in the two villages that immigrated more than 200 km reported a significantly smaller number of forage plants. The long-distance migration of immigrants results in a lack of availability of the biological resources similar to those they had access to in their home area, resulting in changes in the land and biological resource types they have long relied on [52]. For immigrants in environments that lack the biological resources similar to those in their home area, they no longer mention and use these resources; therefore, the specific knowledge associated with these resources will gradually be forgotten [53, 54].

To investigate forage knowledge, the villagers were asked to fully describe the plant parts (e.g., leaves, stems, roots, fruits, or inflorescence) that can be used for feeding livestock [55] and palatability (e.g., amount, juicy texture, sweet taste, bitter taste) [2]. The villagers provided information regarding the different adaptive strategies of feeding cattle and sheep in spring, summer, autumn, and winter [4]. In addition, the details regarding how to use the plants as forage were explained. Two forms, including green tender forage and green hay, were described for further classification of forage plants [56], which is of great value to manual management of forage resources [57]. Particularly, the important effects of some forage plants were summarized [58], which is very valuable. In the interviews, it was learned that some plants can increase fat, act as aphrodisiacs, promote lactation, and reduce “internal heat.” Plants that can significantly increase fat in livestock include *Krascheninnikovia ceratoides*, *Melilotus albus*, *M*. *ruthenica*, *Agriophyllum squarrosum*, *Persicaria vivipara*, *Artemisia gmelinii*, *Artemisia desertorum*, and *Ajania achilleoides.* Plants with aphrodisiac and lactation-promoting effects include the following. The juicy branches of *Artemisia frigida* after the leaves are withered in winter and spring have fattening, fat-retaining, aphrodisiac, and lactation-promoting effects. *Lespedeza davurica*, *Lespedeza potaninii*, and *Glycyrrhiza uralensis* plants can be made into hay at their flowering stage, which serves as good forage for lambing ewes and weak livestock to increase fat and lactation in winter and spring. The medicinal plants with internal heat-reducing and body-strengthening effects are *Rheum tanguticum*, *Rheum palmatum*, and *Rumex crispus*. In summer, these three plants can be boiled in water to produce liquid medicine that can reduce internal heat and relieve summer heat in cattle and sheep. *Corispermum patelliform* plants can be made into highly nutritional hay to feed lambs and sick sheep in winter. The knowledge above was summarized and inherited from long-term production practice and is a precious treasure that deserves to be recorded and studied.

Some villagers reported that the meat from sheep often fed with *Allium mongolicum* and *Allium polyrhizum* tastes better and is free of a mutton smell. Some villagers mentioned that cattle like *Convolvulus arvensis* plants best. As the local folk adage says “Bitter Vine, donkeys don't eat, horses don't watch, old cattle come to pull as long noodles, and goats and sheep love to eat”; donkeys and horses do not like “Bitter Vine” (local name for *C. arvensis*), but cattle like it as much as the local people like long noodles (a kind of pasta that the local people generally like to eat). Several other plants can be used as forage for cattle and sheep only at the early growing period and become toxic and inedible after fruiting, including *Lappula myosotis*, *Lappula squarrosa* subsp. *Heteracantha*, and *Xanthium strumarium* subsp. *sibiricum*. The green tender plants of these three species are good forage for cattle and sheep but become harmful when the seeds mature because the bristles on the edges of the seeds easily stick to the body. In addition, sheep occasionally eats the flowers of *A*. *mongolicus*, as mentioned earlier. However, *A*. *mongolicus* is an important sand-fixing and ancient plant species in semiarid desertified land and a nationally key protected plant. Therefore, villagers should protect this plant from damage during sheep grazing. The knowledge above is of great significance in the development and production of animal husbandry.

Immigrants can make full use of forage plant resources to develop animal husbandry [59], which provides a reliable livelihood for local villagers [60–62]. Cattle and sheep are the main local livestock, playing an important role in the local diet, festivals, weddings, and other customs [59, 62, 63]. Beef and mutton are the main meat sources in the study area and are used to make local delicacies such as boiled lamb, steamed lamb, stewed snacks, stewed beef and lamb soup, soup bowl, stir-fried stewed meat, and sauced beef [59]. Beef and lamb dishes, “Sangza” (deep-fried twisted noodles), “Youxiang” (cake of flour with salt, fried in sesame oil), and stewed noodle soup are necessities at New Year holidays and other festivals [43]. For local marriage customs including the marriage proposal, tea ceremony, flower-adorning ceremony (i.e., place a beautiful flower in the hair of the woman to confirm the engagement), wedding ceremony, and bride’s first home visit after the wedding, sheep are the major gift because they symbolize the success and happiness of the marriage, as said in the local proverb “sheep, wheat, and oil are sent to the bride’s home as gifts between the engagement and wedding.” For each ceremony, the gifts include a sheep, 100 jin of rice (“jin” is a weight unit commonly used in China, and one jin is equal to 0.5 kg), sugar, tea leaves, large steamed buns, each weighing 0.5 jin, and “Youxiang,” etc. [64]. As seen with these customs, raising and breeding cattle and sheep, as well as possessing related forage plant knowledge, are critical in local life. Because of their traditional customs associated with cattle and sheep, the livelihood of ecological immigrants still depends on raising and breeding cattle and sheep even after relocation [65]. Therefore, protecting the traditional culture of immigrants and preserving traditional knowledge associated with biodiversity synergistically promote each other.

Human factors are key for the inheritance of traditional forage plant knowledge. The age, gender, education level, and occupation of ecological immigrants are factors commonly considered in research [66]. In this study, the number of traditional forage plant species reported by respondents was positively correlated with age. Older people could enumerate more forage plant species than could young people, suggesting that elders play a key role in preserving traditional forage plant knowledge and that young people have less understanding of their ancestral homeland after ecological migration. This indicates that there is a disruption risk regarding traditional knowledge inheritance. In the history of local animal husbandry, males have played a more important role and have more knowledge of traditional feeding plants than do females. However, with the livelihood transformation from farming and livestock to non-farming jobs, increasingly more males are pursuing non-farming jobs, and tradition knowledge is gradually being lost. In addition, the number of traditional forage plant species reported by respondents was negatively correlated with education level. The uneducated population (illiterate) had the most abundant knowledge of traditional forage plants, which decreased in the respondents with a higher education level. A possible reason is that uneducated immigrants, who cannot read or write, inherit the traditional knowledge of forage plants through listening and memorizing, while educated immigrants choose non-farming jobs in cities and no longer rely on the traditional farming industry like older generations. This causes a disruption in retaining and passing traditional forage plant knowledge. With regards to occupation, village cadres, knowledgeable masters of local livestock farming, businessmen buying and selling cattle and sheep, and local grassroots doctors have the most knowledge of forage plants and provide key information for the investigation of traditional forage plants. These people play important roles in the protection and inheritance of traditional forage plant knowledge, and they should be supported and helped jointly by the local government, science and technology sectors, forestry and grassland sectors, and industrial associations through incentive measures.

## Conclusions

This study investigated traditional forage plant species and associated traditional knowledge learned and mastered by the villagers who moved to four ecological immigrant villages and those who still live in two villages near the emigration area in the Hongsibu District of Ningxia. Detailed information regarding 224 traditional forage plants were provided, including local names, plant parts commonly used as forage, forage form, and livestock for which the plant is suitable to feed. The value and utilization knowledge of local important forage plants were analyzed. In addition, the differences in the types and number of traditional forage plant species and related knowledge reported during the survey were compared among the ecological immigrants from different areas. Moreover, the effects of gender, age, education level, and occupation on traditional forage plant knowledge were analyzed. We concluded that forage plant resources in the ecological immigration area surveyed is highly diverse and abundant, and forage plant species vary among the forest areas, loess hilly areas, and semiarid desertified areas. Due to different biodiversity backgrounds and living environments, there are differences in the diversity of traditional forage plants and related knowledge retained by ecological immigrants from different emigration areas. Immigrants who move only a short distance well retain the diversity of traditional forage plant sources and related knowledge. In contrast, immigrants who migrate a long distance experience remarkable changes in natural resources and thus have less knowledge regarding the traditional forage plants at the new location. The knowledge of forage plants is being forgotten and abandoned by the younger generation. The changes above may be attributed to multiple factors including changes in the distribution of forage plant resources, livestock farming traditions, and customs. Males, elders, people with less education, and key figures in the villages know the most about traditional forage plants. We suggest that targeted measures should be taken to record and protect forage plant resources and knowledge retained and passed on by ecological immigrants, promoting regional biodiversity preservation and sustainable development.

## Data Availability

The analyzed data are incorporated in the research article.
